# Assessing gene-environment interaction effects of FTO, MC4R and lifestyle factors on obesity using an extreme phenotype sampling design: Results from the HUNT study

**DOI:** 10.1371/journal.pone.0175071

**Published:** 2017-04-06

**Authors:** Thea Bjørnland, Mette Langaas, Valdemar Grill, Ingrid Løvold Mostad

**Affiliations:** 1 Department of Mathematical Sciences, Faculty of Information Technology and Electrical Engineering, Norwegian University of Science and Technology, Trondheim, Norway; 2 Department of Cancer Research and Molecular Medicine, Faculty of Medicine and Health Sciences, Norwegian University of Science and Technology, Trondheim, Norway; 3 Department of Endocrinology, St. Olavs Hospital, Trondheim University Hospital, Trondheim, Norway; 4 Department of Clinical Nutrition and Speech-Language Therapy, Clinic of Clinical Services, St. Olavs Hospital, Trondheim University Hospital, Trondheim, Norway; Children's National Health System, UNITED STATES

## Abstract

**Background:**

Our aim was to assess the influence of age, gender and lifestyle factors on the effect of the obesity-promoting alleles of FTO and MCR4.

**Methods:**

The HUNT study comprises health information on the population of Nord-Trøndelag county, Norway. Extreme phenotype participants (gender-wise lower and upper quartiles of waist-hip-ratio and BMI ≥ 35 kg/m^2^) in the third survey, HUNT3 (2006–08), were genotyped for the single-nucleotide polymorphisms rs9939609 (FTO) and rs17782313 (MC4R); 25686 participants were successfully genotyped. Extreme sampling was chosen to increase power to detect genetic and gene-environment effects on waist-hip-ratio and BMI. Statistical inference was based on linear regression models and a missing-covariate likelihood approach for the extreme phenotype sampling design. Environmental factors were physical activity, diet (artificially sweetened beverages) and smoking. Longitudinal analysis was performed using material from HUNT2 (1995–97).

**Results:**

Cross-sectional and longitudinal genetic effects indicated stronger genetic associations with obesity in young than in old, as well as differences between women and men. We observed larger genetic effects among physically inactive compared to active individuals. This interaction was age-dependent and seen mainly in 20–40 year olds. We observed a greater FTO effect among men with a regular intake of artificially sweetened beverages, compared to non-drinkers. Interaction analysis of smoking was mainly inconclusive.

**Conclusions:**

In a large all-adult and area-based population survey the effects of obesity-promoting minor-alleles of FTO and MCR4, and interactions with life style factors are age- and gender-related. These findings appear relevant when designing individualized treatment for and prophylaxis against obesity.

## Introduction

It is well recognized that both genetic and non-genetic factors are operative in the development and persistence of obesity. Polymorphisms of the FTO gene are shown to be the strongest genetic determinant of obesity [1] compared with the > 40 other genes which affect body weight [2, 3]. Given the primacy of FTO, the minor-allele exerts only modest effects in most studies. It is possible that interactions with other factors may under some conditions lead to an increase (or decrease) in effects. However, whether the impact of FTO on obesity is modified by lifestyle factors, such as diet, physical activity and smoking has only been partly elucidated. Also gene-environment interactions should be tested in relation to age and gender, something that has so far only been rarely done.

Interactions with physical activity have been demonstrated when results from a large number of different populations have been pooled together [4, 5]. However, such meta-analyses have also revealed differences between populations [5], something which is also evident in individual studies, some demonstrating an interaction [6–8] others not [9]. Such differences reduce the possibilities to characterize an interaction in detail. A large individual study could then be preferable for detailed characterization. This also applies to diet where results are discordant between investigations as to interactions with FTO [10–15], and non-conclusive as to smoking [16]. A study encompassing different ages and both genders would also allow the assessment of whether age and gender modify interactions with the lifestyle factors mentioned. The potential importance of age and gender for FTO effects was previously recognised [17, 18] but the effects have so far been insufficiently characterized.

The HUNT all-population survey provides data that appear to be representative of Norway and Scandinavia [19]. Here we designed a study for the associations between FTO (rs9939609) and obesity, including gene-environment interactions, based on data from HUNT3 (2006–2008). Results on FTO were compared with results on MCR4 (rs17782313), which is also an influential gene [20], albeit not with the same impact as FTO in relation to obesity [21]. We previously analysed waist-hip-ratio (WHR) with respect to a range of dietary variables in the HUNT material [22]. Here, we obtained genotypes for approximately half of the HUNT3 participants by selecting individuals according to an extreme phenotype sampling design (extremes being lowest and highest quartiles of WHR, and additionally all individuals with BMI ≥ 35 kg/m^2^), rather than drawing a random sample. Genotyping the phenotypic extremes is considered to increase the statistical power to detect associations between genetic variants and continuous phenotypes [23]. In addition to large sample sizes, choosing a powerful design can be particularly important for analysis of gene-environment interactions (GEIs), which one would expect to have low power to detect [24–26]. By genotyping individuals with extreme WHR and BMI values, and by carefully selecting and constructing environmental variables from the HUNT material we should have good power to detect GEIs. Statistical inference methods that correctly account for extreme sampling have been proposed in the statistical literature [23], but to our knowledge not applied to the extent that we have done here. By adapting these methods, we have assessed over-all impact as well as gene-environment interactions with respect to WHR and BMI. Analysis was performed gender-wise in three age groups. Although most of the data analysed were cross-sectional, we also assessed longitudinal effects, then using data from HUNT2 (1995–97).

## Materials and methods

The HUNT study [19] comprises a database of health and medical information on the population of Nord-Trøndelag county, Norway, collected in three surveys; HUNT1 (1984–86), HUNT2 (1995–97) and HUNT3 (2006–08). In HUNT3 the participation rate out of the 93860 invited was 54.1%. Trained nurses performed measurements of waist circumference, hip circumference, weight and height. From these measurements we calculated WHR and BMI. We carefully constructed three environmental lifestyle variables based on Questionnaire 1 in HUNT3 to represent physical activity, diet and smoking. We summarized four questions on physical activity into a 9-leveled index. The index then captured frequency, intensity and duration of exercise, in addition to overall daily physical inactivity. Four questions on cigarette smoking were summarized in the variable of pack years, defined as the average number of packs of 20 cigarettes smoked per year during the period of daily smoking. Mostad *et al* has previously analysed the relationship between WHR and dietary variables in HUNT3 [22]. Here we selected artificially sweetened beverages (measured in glasses drunk per week) as a dietary explanatory variable because artificially sweetened beverages was found to be strongly associated with WHR (57% higher intake among individuals with central obesity compared to individuals without central obesity [22]). For details on the construction of environmental lifestyle variables from Questionnaire 1, see S1 Appendix.

Extreme phenotype participants in HUNT3 were genotyped for FTO (rs9939609) and MC4R (rs17782313). Extreme phenotypes were defined as the gender-wise lower and upper quartiles of WHR (WHR < 0.817 and WHR > 0.917 for women, WHR < 0.895 and WHR > 0.981 for men) and additionally BMI ≥ 35 kg/m^2^. Of the 25981 extreme phenotype individuals, 25686 were successfully genotyped for both SNPs (98.9%). See S1 Appendix for details on the genotyping procedure.

The Regional Committees for Medical and Health Research Ethics for central Norway (REC Central) approved this research project.

### Statistical methods

We assumed that in the underlying population, WHR and BMI could be modelled with linear regression models with environmental covariates (age, physical activity (PA), artificially sweetened beverages (ASB) and smoking (PCYR)) and genetic covariates (FTO, MC4R). Age was coded into 5-year intervals and treated as a categorical variable, denoted *x*_*age*_. The physical activity, diet and smoking variables were treated as continuous explanatory variables, *x*_*PA*_, *x*_*ASB*_ and *x*_*PCYR*_. We analysed data from men and women separately. Genetic covariates were genotypes of the FTO and MC4R SNPs, *x*_*FTO*_ and *x*_*MC4R*_ and coded as 0, 1 or 2 according to the number of copies of the minor-allele (FTO: 0 = TT, 1 = TA, 2 = AA, MC4R: 0 = TT, 1 = TC, 2 = CC). Our basic regression model for over-all genetic effects was

Y=η+ε (1)

where *Y* is a trait (WHR or BMI), ε is normally distributed with mean 0 and variance σ^2^, and

$$\eta =\;\alpha +\beta_{age}x_{age}+\beta_{PA}x_{PA}+\beta_{ASB}x_{ASB}+\beta_{PCYR}x_{PCYR}+\beta_{FTO}x_{FTO}+\beta_{MC4R}x_{MC4R}$$

For longitudinal analysis, we modelled the difference in traits Δ*Y* between HUNT3 and HUNT2 by

ΔY=η+ε (2)

where η is as in Model (1). All lifestyle and genetic covariates were taken from HUNT3 while changes in WHR and BMI were calculated based on HUNT2 and HUNT3 data. Longitudinal analysis was then based on individuals participating in both HUNT2 and HUNT3. For gene-environment interactions we included a statistical interaction term between the relevant environmental covariate and SNPs and thereby assessed departures from additive effects. For example for interactions with artificially sweetened beverages the regression model was

$$Y=\eta +\;\beta_{FTO*ASB}x_{FTO}x_{ASB}+\;\beta_{MC4R*ASB}x_{MC4R}x_{ASB}+\varepsilon$$ (3)

where *η* is as in Model (1), and similarly for physical activity and smoking. In the following, the term “estimated effect of FTO” refers to estimates of the parameter *β*_*FTO*_ (over-all or longitudinal effect) while the term “estimated GEI effect” refers to estimates of *β*_FTO*ENV_ (where *ENV* is any of the three environmental variables), and similarly for MC4R.

By the extreme sampling design the variables *y*, *x*_*age*_, *x*_*PA*_, *x*_*ASB*_ and *x*_*PCYR*_ were observed for the full sample, while the genetic variables *x*_*FTO*_ and *x*_*MC4R*_ were only observed for the extreme phenotype individuals. The genetic covariates were then missing at random in the full sample, and we used likelihood methods for missing covariate data (S2 Appendix). In such missing covariate likelihoods, the distribution of the missing covariates (here FTO and MC4R) must be estimated. We assumed that SNP genotypes could be modelled by multinomial distributions that were independent of all non-genetic covariates. We verified this assumption by testing for independence between genotypes and environmental covariates in the extreme genotyped samples, and by sensitivity analyses (S2 Appendix). Maximum likelihood estimates of parameters in the three regression models were found by numerical optimization. We implemented the score test for extreme sampling data to test *H*_0_: *β*_*FTO*_ = 0 against *H*_1_: *β*_*FTO*_ ≠ 0, and *H*_0_: *β*_*MC*4*R*_ = 0 against *H*_1_: *β*_*MC*4*R*_ ≠ 0 in the over-all effects Model (1) and the longitudinal Model (2). We implemented the likelihood ratio test for extreme sampling data to test GEIs such as *H*_0_: *β*_FTO*PA_ = 0 against *H*_1_: *β*_FTO*PA_ ≠ 0 in Model (3). All statistical methods were implemented and executed in R [27], see S2 Appendix for further details.

An important assumption for linear regression models is independence. The HUNT3 population is a stable population; hence many individuals should be related. Due to privacy regulations any familial ties between study participants were unknown to us. We divided the sample into age groups; 20–40 years (20 ≤ age < 40), 40–60 years (40 ≤ age < 60), 60–80 years (60 ≤ age < 80) that we analysed separately. This should eliminate to a large extent the unknown but assumed co-presence of parents and children in each sample. Importantly, such stratified analysis also allowed us to detect age- and gender-related influences on the genetic impact of FTO and MCR4.

A large number of tests were performed in our analysis because we analysed six age and gender groups separately for two outcomes (WHR and BMI). For each of these, we considered over-all effects, longitudinal effects and three different GEIs; in total 120 tests. To account for the large number of tests, we have chosen to control the false discovery rate (FDR) at the 5% level using the Benjamini-Hochberg step-up procedure [28].

#### Power-simulation study

We performed a power simulation study to verify that the extreme sampling design could in fact be more powerful than a random sampling design for our analysis (S3 Appendix). We used all non-genetic covariates from the 20–40 year female stratum, and then simulated a genetic covariate. We used the actual parameter estimates from this stratum and simulated a new response. Then the estimated statistical power to detect a non-zero GEI effect at significance level 0.05 was 84% in the full (simulated) data set. We then considered genotyping only the extremes (upper and lower quartile), compared to genotyping a random sample. We found that the extreme sampling design had 80% power, while a random sampling design had 56% power. Thus, to detect GEIs in our data when a fixed number of individuals (approximately half of all HUNT3 participants) could be genotyped, it seems crucial to select those with extreme phenotypes, rather than a random sample.

## Results

**Fig 1:**
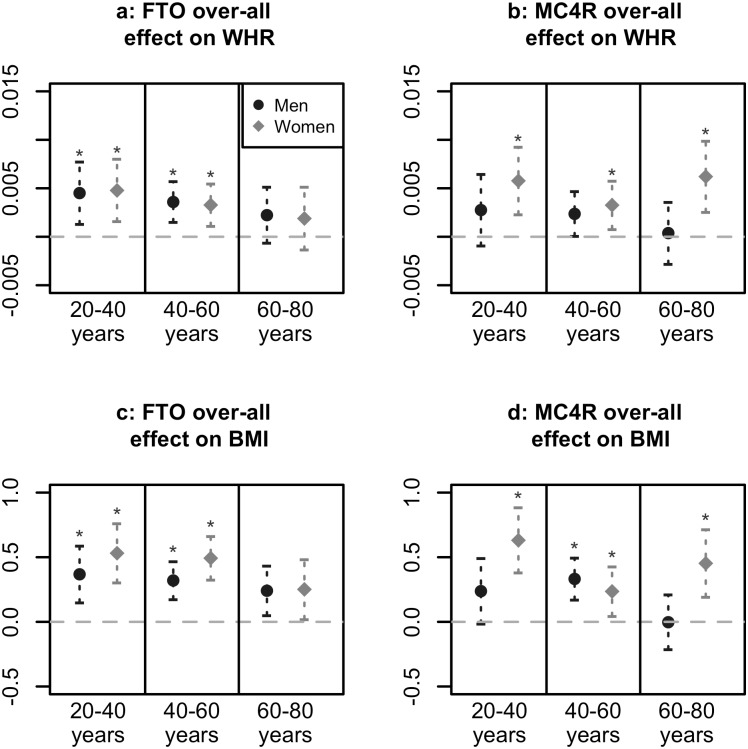
Over-all genetic effects. Over-all effects of FTO and MC4R with 95% confidence intervals, for men and women in three age groups. Asterisk indicates FDR-adjusted p-value below 0.05 for testing *H*_0_: *β*_*SNP*_ = 0 against *H*_1_: *β*_*SNP*_ ≠ 0.

**Fig 2:**
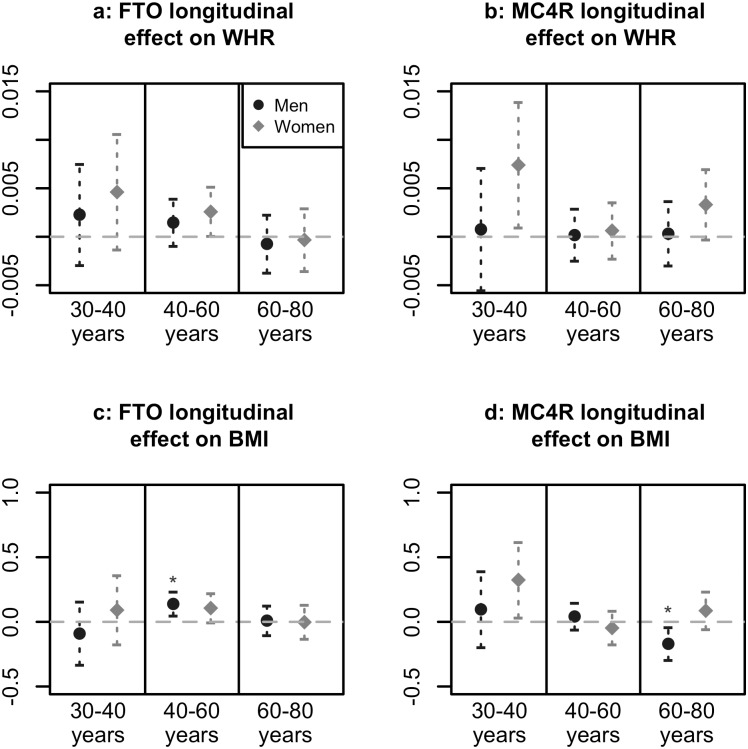
Longitudinal genetic effects. Longitudinal effects of FTO and MC4R with 95% confidence intervals, for men and women in three age groups (30–40 years as youngest age group because individuals younger than 30 years in HUNT3 did not participate in HUNT2). Asterisk indicates FDR-adjusted p-value below 0.05 for testing *H*_0_: *β*_*SNP*_ = 0 against *H*: *β*_*SNP*_ ≠ 0.

Relevant sample sizes for our analysis are presented in Table 1. The age and gender groups varied in size between 3000 and 8000 participants, the largest being the 40–60 years age group. Estimates of *β*_*FTO*_ and *β*_*MC4R*_ in the over-all effects Model (1) and longitudinal effects Model (2) are presented Figs 1 and 2 and in S1 and S2 Tables. In S1 and S2 Tables we also report FDR-adjusted p-values for the hypothesis tests *H*_0_: *β*_*FTO*_ = 0 against *H*_1_: *β*_*FTO*_ ≠ 0, and *H*_0_: *β*_*MC*4*R*_ = 0 against *H*_1_: *β*_*MC*4*R*_ ≠ 0. Estimates of GEIs in Model (3), and adjusted p-values for the hypothesis tests *H*_0_: *β*_FTO*ENV_ = 0 and *H*_0_: *β*_*MC*4R*ENV_ = 0 are presented in Table 2 (physical activity), Table 3 (artificially sweetened beverages) and Table 4 (pack years of smoking). Out of the 120 tests for association that we performed, 24 tests had FDR-adjusted p-values below 0.05. Thus, by controlling the false discovery rate at a 0.05 significance level we have 24 findings. Below we summarize main findings based on both estimated effect sizes and significance of test results.

**Table 1:** Sample sizes.

	20–40 years		40–60 years		60–80 years	
	Men	Women	Men	Women	Men	Women
**Complete sample**	3237	4817	6694	7944	4084	3948
**Genotyped sample**	2029	2740	3081	3794	1925	2037
**HUNT2/HUNT3 sample**	993*	1505*	5216	6397	3693	3660

Sample sizes gender-wise for each age group, 20–40 years (*30–40 years in HUNT2-HUNT3 analysis because individuals younger than 30 years in HUNT3 did not participate in HUNT2), 40–60 years and 60–80 years. Genotyped sample refers to individuals who were genotyped based on the extreme phenotype criteria.

**Table 2:** Interactions with physical activity.

Trait	SNP	Age group	Gender	*β*_*PA*_	*β*_*SNP*_	*β*_SNP*PA_	95% CI for *β*_SNP*PA_	Adjusted p-value
WHR	FTO	20–40 years	Men	-0.0026	0.017	-0.0029	-0.0048, -0.0011	0.029
			Women	6.9e-05	0.017	-0.0028	-0.0048, -0.00073	0.048
		40–60 years	Men	-0.0066	0.0024	0.00028	-0.001, 0.0016	0.86
			Women	-0.0038	0.0018	0.00034	-0.0012, 0.0019	0.86
		60–80 years	Men	-0.007	0.0044	-0.00051	-0.0023, 0.0013	0.81
			Women	-0.0088	-0.00081	0.00064	-0.0017, 0.003	0.86
	MC4R	20–40 years	Men	-0.0026	0.0085	-0.0013	-0.0034, 0.00071	0.51
			Women	6.9e-05	0.01	-0.001	-0.0032, 0.0012	0.7
		40–60 years	Men	-0.0066	0.0042	-0.00043	-0.0019, 0.0011	0.84
			Women	-0.0038	0.0051	-0.00043	-0.0022, 0.0013	0.86
		60–80 years	Men	-0.007	-0.0066	0.0016	-0.00033, 0.0036	0.3
			Women	-0.0088	0.0068	-0.00014	-0.0027, 0.0025	1
BMI	FTO	20–40 years	Men	-0.041	1.0	-0.15	-0.28, -0.018	0.11
			Women	0.053	1.4	-0.2	-0.34, -0.049	0.048
		40–60 years	Men	-0.22	0.36	-0.0088	-0.1, 0.082	1
			Women	-0.30	0.84	-0.078	-0.2, 0.04	0.45
		60–80 years	Men	-0.40	-0.078	0.075	-0.043, 0.19	0.46
			Women	-0.66	0.14	0.027	-0.14, 0.19	0.95
	MC4R	20–40 years	Men	-0.041	0.26	-0.0075	-0.15, 0.14	1
			Women	0.053	1.7	-0.24	-0.41, -0.078	0.025
		40–60 years	Men	-0.22	0.46	-0.031	-0.13, 0.073	0.81
			Women	-0.3	0.024	0.048	-0.085, 0.18	0.78
		60–80 years	Men	-0.4	-0.6	0.14	0.015, 0.27	0.11
			Women	-0.66	0.64	-0.046	-0.23, 0.14	0.86

Gene-environment interaction effects for physical activity (PA); estimated main effect of physical activity (*β*_*PA*_), estimated main effect of SNPs (*β*_*SNP*_), estimated GEI effects (*β*_SNP*PA_), 95% confidence intervals for the GEI effects and FDR-adjusted p-value for testing *H*_0_: *β*_SNP*PA_ = 0 against *H*_0_: *β*_SNP*PA_ ≠ 0.

**Table 3:** Interactions with artificially sweetened beverages.

Trait	SNP	Age group	Gender	*β*_*ASB*_	*β*_*SNP*_	*β*_SNP*ASB_	95% CI for *β*_SNP*ASB_	Adjusted p-value
WHR	FTO	20–40 years	Men	0.0013	0.0029	0.00037	-0.00018, 0.00091	0.43
			Women	0.0013	0.005	-4.3e-05	-0.00056, 0.00047	1
		40–60 years	Men	0.0011	0.0024	0.00043	-6.1e-05, 0.00093	0.43
			Women	0.0012	0.0025	3e-04	-2e-04, 0.00081	0.66
		60–80 years	Men	8e-04	0.00059	0.00094	6.3e-05, 0.0018	0.15
			Women	0.0015	0.0013	0.00032	-0.00067, 0.0013	0.87
	MC4R	20–40 years	Men	0.0013	0.0046	-0.00041	-0.001, 0.00019	0.43
			Women	0.0013	0.0051	0.00013	-0.00043, 7e-04	0.89
		40–60 years	Men	0.0011	0.0028	-0.00016	-0.00072, 0.00039	0.72
			Women	0.0012	0.0026	0.00027	-3e-04, 0.00084	0.72
		60–80 years	Men	8e-04	0.0017	-7e-04	-0.0016, 0.00018	0.19
			Women	0.0015	0.0068	-0.00042	-0.0015, 7e-04	0.72
BMI	FTO	20–40 years	Men	0.085	0.13	0.059	0.021, 0.097	0.024
			Women	0.14	0.52	0.002	-0.037, 0.041	1
		40–60 years	Men	0.056	0.19	0.053	0.019, 0.086	0.024
			Women	0.17	0.46	0.013	-0.025, 0.052	0.81
		60–80 years	Men	0.074	0.15	0.059	-0.00049, 0.12	0.15
			Women	0.19	0.35	-0.069	-0.14, 0.0046	0.2
	MC4R	20–40 years	Men	0.085	0.39	-0.038	-0.082, 0.0049	0.22
			Women	0.14	0.62	0.0028	-0.041, 0.046	1
		40–60 years	Men	0.056	0.33	-0.00019	-0.039, 0.039	1
			Women	0.17	0.3	-0.024	-0.067, 0.02	0.59
		60–80 years	Men	0.074	0.044	-0.021	-0.08, 0.038	0.62
			Women	0.19	0.41	0.025	-0.064, 0.11	0.78

Gene-environment interaction effects for artificially sweetened beverages (ASB; estimated main effect of artificially sweetened beverages (*β*_*ASB*_), estimated main effect of SNPs (*β*_*SNP*_), estimated GEI effects (*β*_SNP*ASB_), 95% confidence intervals for the GEI effects and FDR-adjusted p-value for testing *H*_0_: *β*_SNP*ASB_ = 0 against *H*_1_: *β*_SNP*ASB_ ≠ 0.

**Table 4:** Interactions with pack years.

Trait	SNP	Age group	Gender	*β*_*PCYR*_	*β*_*SNP*_	*β*_SNP*PCYR_	95% CI for *β*_SNP*PCYR_	Adjusted p-value
WHR	FTO	20–40 years	Men	0.001	0.0044	2e-05	-0.00071, 0.00075	1
			Women	0.0016	0.0039	0.00042	-0.00036, 0.0012	0.59
		40–60 years	Men	0.00045	0.0013	3e-04	0.00011, 0.00048	0.81
			Women	0.00079	0.0018	0.00023	-8.8e-06, 0.00046	1
		60–80 years	Men	0.00036	-3.2e-5	0.00019	1.7e-05, 0.00036	1
			Women	-0.00011	-0.0015	0.00036	7.3e-05, 0.00065	0.73
	MC4R	20–40 years	Men	0.001	0.0017	5e-04	-0.00035, 0.0013	0.5
			Women	0.0016	0.0057	-3.3e-06	-0.00078, 0.00077	1
		40–60 years	Men	0.00045	0.0022	1.8e-05	-0.00019, 0.00023	1
			Women	0.00079	0.0042	-0.00013	-0.00039, 0.00013	0.42
		60–80 years	Men	0.00036	-0.0015	0.00014	-2.9e-05, 0.00031	1
			Women	-0.00011	0.0063	-7e-05	-0.00039, 0.00025	1
BMI	FTO	20–40 years	Men	0.076	0.37	0.00098	-0.051, 0.053	1
			Women	0.018	0.4	0.063	0.0056, 0.12	0.1
		40–60 years	Men	0.0012	0.2	0.016	0.0041, 0.028	0.048
			Women	0.033	0.63	-0.019	-0.036, -0.0023	0.1
		60–80 years	Men	0.023	0.31	-0.0053	-0.016, 0.0055	0.73
			Women	-0.036	0.11	0.02	0.0015, 0.039	0.11
	MC4R	20–40 years	Men	0.076	0.24	-0.0045	-0.066, 0.057	1
			Women	0.018	0.56	0.026	-0.03, 0.083	0.5
		40–60 years	Men	0.0012	0.34	-0.0014	-0.015, 0.012	1
			Women	0.033	0.37	-0.018	-0.037, 0.00021	0.15
		60–80 years	Men	0.023	0.099	-0.0078	-0.018, 0.0026	0.42
			Women	-0.036	0.54	-0.011	-0.033, 0.011	0.5

Gene-environment interaction effects for pack-years (PCYR); estimated main effect of smoking (*β*_*PCYR*_), estimated main effect of SNPs (*β*_*SNP*_) and estimated GEI effects (*β*_SNP*PCYR_), 95% confidence intervals for the GEI effects and FDR-adjusted p-value for testing *H*_0_: *β*_SNP*PCYR_ = 0 against *H*_1_: *β*_SNP*PCYR_ ≠ 0.

### Over-all genetic effects

Over-all effects of the FTO and MC4R SNPs on WHR are presented in Fig 1. The FTO minor-allele was associated with higher WHR in 20–40 and 40–60 year olds (Fig 1a). For both genders, the effect sizes of FTO tended to be highest in the youngest age group. Similar results were seen for BMI (Fig 1c). The MC4R minor-allele was associated with higher WHR in all female age groups (Fig 1b), and with BMI also in the male 40–60 year olds (Fig 1d). Among women the greatest effect sizes of MC4R were found among 20–40 and 60–80 year olds while in men the association decreased with age.

### Longitudinal effects

In the HUNT population, average WHR and BMI generally increased in the decade between HUNT2 and HUNT3 in all age and gender groups (S3 Table). Our hypothesis was then that this increase was more (or less) rapid depending on the FTO or MC4R genotype. Estimated longitudinal effects of FTO and MC4R on WHR and BMI are presented in Fig 2. Two significant associations (FDR-adjusted p-value < 0.05) were found; the FTO minor-allele was associated with a more rapid increase in BMI in 40–60 year old men, and the MC4R minor-allele was associated with a less rapid increase in BMI among 60–80 year old men. Although significantly different from zero, these effect sizes are negligible compared to the over-all effects presented in Fig 1. Sample sizes were low for the youngest HUNT3 individuals (Table 1), but longitudinal effect size estimates and confidence intervals indicated that the MC4R minor-allele was associated with a more rapid increase in WHR and BMI in young women between HUNT2 and HUNT3 (Fig 2b and 2d). These effect sizes are comparable to the over-all effects.

### Interactions with physical activity

**Fig 3:**
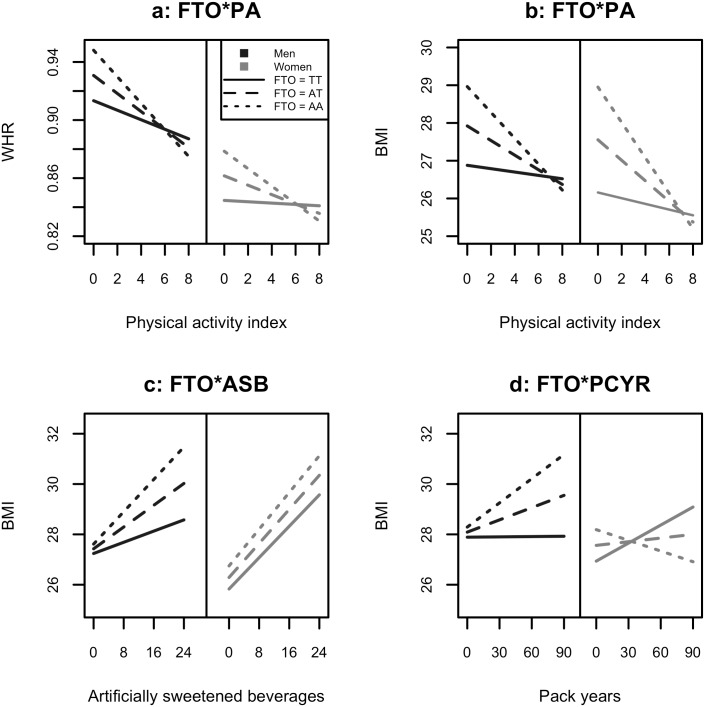
Gene-environment interaction effects. Estimated WHR (a) and BMI (b) in the 20–40 years age group for all FTO genotypes and for increasing levels of physical activity (PA) for an average man (age 31, ASB 4.2, PCYR 1.8) and woman (age 31, ASB 4.7, PCYR 2.1). Estimated BMI in the 40–60 years age group for all FTO genotypes for increasing levels of intake of artificially sweetened beverages (c) and increasing number of pack years (d) for an average man (age 51, PA 4.2, ASB 2.7, PCYR 7.4) and woman (age 49, PA 4.3, ASB 2.7, PCYR 7.1).

Estimated parameters from modelling interaction effects between FTO and MC4R and physical activity are presented in Table 2. For both men and women 20–40 years, we found that the interaction between physical activity and FTO was associated with WHR (FDR-adjusted p-value < 0.05). For women 20–40 years, we also found that both the interactions between FTO and physical activity, and MC4R and physical activity were associated with BMI. Consider a man 20–40 years with one copy of the FTO minor-allele. In our regression model for WHR with interactions (Model (3)) we estimated *β*_*FTO*_ = 0.017 and *β*_FTO*PA_ = −0.0029 (Table 2). Thus, with physical activity index 5 or greater, we see that the effect of one FTO minor-allele was cancelled out by the interaction between FTO and physical activity. In Fig 3a we have plotted estimated WHR against increasing levels of physical activity for an average man and woman 20–40 years for the three genotypes of FTO. Similarly, estimated BMI is presented in Fig 3b. Estimated WHR or BMI was quite similar for individuals with high levels of physical activity, regardless of FTO genotype. For individuals with low levels of physical activity, those with one or two copies of the FTO minor-allele had higher estimated WHR or BMI than those with no minor-allele. In other words, the over-all effect of FTO on obesity was negligible in highly active individuals aged between 20 and 40 years.

### Interactions with artificially sweetened beverages

Estimated parameters from modelling interaction effects between FTO and MC4R and weekly intake of artificially sweetened beverages are presented in Table 3. Among men, aged 20–40 and 40–60 years, the interaction between artificially sweetened beverages and FTO was significantly associated with BMI. Estimated BMI in the 40–60 year age group (for an average man and woman) for all genotypes of FTO and with respect to increasing intake of such beverages is presented in Fig 3c. Among both men and women the estimated BMI increased as the weekly intake of artificially sweetened beverages increased. In men, the increase was more rapid in individuals with one or more copies of the FTO minor-allele. In other words, a high intake of artificially sweetened beverages was associated with a higher BMI in men with the FTO minor-allele, compared to men with no FTO minor-allele. Among individuals with a low intake of such beverages, the estimated BMI was similar for all men, regardless of FTO genotype. In women, no significant GEI effect was found and estimated BMI increased with the same rate for increasing intake, regardless of FTO genotype.

### Interactions with smoking (pack years)

Estimated parameters from modelling interaction effects between FTO and MC4R and pack years of smoking are presented in Table 4. The direction of the over-all smoking effects and GEI effects tended to vary between age groups, genders and between WHR and BMI.

For men 40–60 years, the interaction between smoking and FTO was associated with increased BMI. We estimated *β*_*FTO*_ = 0.201 and *β*_FTO*PCYR_ = 0.016. For women 40–60 years the direction of the GEI effect seemed the opposite of that found in men (Fig 3d) but was not significant. Here, for men, estimated BMI was not affected by smoking in individuals with no FTO minor-alleles, while for men with one or more FTO minor-allele, an increase in pack years was associated with higher BMI. For women, estimated BMI increased with pack years for individuals with no FTO minor-allele, but decreased with pack years for individuals with two copies of the FTO minor-allele. In women 20–40 and 60–80 years, the estimated GEI effect was in the opposite direction of that found in women 40–60 years (Table 4).

## Discussion

Here we have assessed gene-environment interaction effects of FTO and MC4R on obesity, taking into account any modulation due to age and gender. Our contribution to this field seems important since our data material comes from a large homogeneous population- and area-based study (HUNT). Anthropometric measurements in HUNT were taken by trained nurses and not self-reported. We have carefully selected environmental covariates to represent various lifestyle aspects: physical activity, smoking and diet. Furthermore we used the extreme phenotype sampling design in order to increase the statistical power of our study, as compared to randomly sampling study participants.

We expectedly confirm an overall obesity-promoting effect of the FTO and MC4R minor-alleles in our study population. Of note, the effect appeared age-dependent with the strongest effect seen in the 20–40 year olds. Also our longitudinal findings indicate a diminished or absent influence of the FTO and MC4R minor-alleles at older age. An age-dependent attenuation of the FTO effect in adults has also been reported in other populations, albeit in smaller [17] and non-population-based [18] studies which did not include the highest age group of the present study [29–32]. (Some studies indicate a biphasic development with increasing effect during childhood and adolescence, followed by decreasing influence. Our study population did not include non-adults). In our analysis, MC4R effects seemed more pronounced in women than in men. Gender related effects on the impact of genetic factors on obesity have also previously been recognized [17, 18] but not characterized in detail. Gender-related differences can tentatively be ascribed to hormonal influences and gender-dependent differences in regional depots of adipose tissue in which the regulation of metabolism is known to differ [33, 34], and on which genetic influences may vary [35–37].

Genetic interactions with physical activity and in particular the age- and gender-dependent modulations of these effects were the main findings of our study. A previous study based on HUNT data did not find an interaction [38], possibly due to a much smaller study population than the present one. Also, we constructed here a variable to represent physical activity that accounted for frequency, intensity and duration of exercise, in addition to overall daily physical inactivity. Importantly, significant interactions with physical activity were observed only in the 20–40 year age group. In this group, the interaction between FTO and physical activity was associated with WHR and BMI in both genders, and the interaction between MC4R and physical activity was associated with BMI in women. Our results indicate that high physical activity diminishes the obesity-promoting effect of FTO and MC4R. From these results it could seem possible that interactions with physical activity can be generalized to the totality of the genetic load predisposing for obesity. However, the modulating influences of factors such as age and gender may differ between genetic risk factors as further highlighted by our results on diet and smoking, which are discussed below.

For interactions with diet we chose a somewhat unconventional parameter, namely weekly intake of artificially sweetened beverages. The choice of this parameter was based on its strong association with central obesity, as reported by us [22]. Other studies have also reported an obesity-promoting effect of artificially sweetened beverages [39–41]. Reverse causality, i.e. increased intake of artificially sweetened beverages in obese people as an attempt to decrease weight, could certainly be at play. That we find an interaction effect with FTO in men 20–40 and 40–60 years old indicates that the over-all effect of drinking artificially sweetened beverages is at least not wholly explained by reverse causality. In support of this notion several recent studies report increasing appetite [42, 43] and obesity-favouring microbiotic effects [44] by artificially sweetened beverages. We speculate that a likely effect of FTO on food intake may synergize with an effect of artificially sweetened beverages on appetite. In our data material, artificially sweetened beverages was the dietary variable that best described central obesity. It was therefore selected as the variable that would carry most power to detect gene-diet interactions. A more powerful study for gene-diet interactions could perhaps be based on a dietary variable combining several aspects of participants’ diet. In this context it should be recognized as a limitation that all dietary data from HUNT are based on self-reporting.

Smoking has well recognized effects on body size, including associations to obesity. It was therefore of interest to test for interactions between smoking and genetic variants with respect to obesity. We found little evidence of interactions (only in men 40–60 years old), and effect sizes were in varying directions between age groups and genders. Smoking behaviour may differ between the genders and age groups with regard to cigarette brand choices, daily vs. occasional smoking, and association or not with intake of alcoholic beverages; such putative differences may impact the present results. Also, the diverging effects of smoking on body size should be taken into account when interpreting our results; a decreasing effect due to effects of nicotine on metabolism and appetite, increasing effects due to other factors [45]. The pack year variable was chosen to capture the totality of the smoking load and is widely used in epidemiological research on tobacco. Self-reporting is however an obvious limitation for this variable.

Interpretations of our results are subject to limitation by the sizable number of non-participants. The total rate of non-participation in the HUNT3 survey was 46%. Data on non-participants has been assembled and analyzed [46] and the analysis indicates that obesity was more prevalent in non-participants than in participants. Non-participation combined with increased obesity could indicate a different impact of lifestyle factors vs. genetic factors in non-participants than in the present study population.

In conclusion, the present data from an all-adult population survey demonstrate that age and gender importantly modify interactions between lifestyle factors and the obesity-promoting effects of FTO and MCR4. These findings appear relevant when designing individualized treatment for and prophylaxis against obesity.

## Supporting information

S1 Appendix Details on environmental and genetic variables from the HUNT data. (PDF)

S2 Appendix Statistical methods for the extreme phenotype sampling design. (PDF)

S3 Appendix Power simulation study. (PDF)

S1 Table Over-all genetic effects. Estimated over-all effects (*β*_*SNP*_) with 95% confidence intervals for men and women in age groups 20–40 years, 40–60 years and 60–80 years. FDR-adjusted p-values for testing *H*_*0*_: *β*_*SNP*_ = 0 against *H*_*1*_: *β*_*SNP*_ ≠ 0.
(PDF)

S2 Table Longitudinal genetic effects. Estimated longitudinal effects (*β*_*SNP*_) with 95% confidence intervals for men and women in age groups 30–40 years (individuals younger than 30 years in HUNT3 did not participate in HUNT2), 40–60 years and 60–80 years. FDR-adjusted p-values for testing *H*_*0*_: *β*_*SNP*_ = 0 against *H*_*1*_: *β*_*SNP*_ ≠ 0.
(PDF)

S3 Table Trait differences HUNT2 and HUNT3. Average differences in WHR and BMI between HUNT3 and HUNT2 for men and women in three age groups. Positive differences indicate increase from HUNT2 to HUNT3.
(PDF)
