# Supplementary material for: Assessing gene-environment interaction effects of FTO, MC4R and lifestyle factors on obesity using an extreme phenotype sampling design: Results from the HUNT study
Source: PLoS One. 2017 Apr 6;12(4):e0175071. doi: 10.1371/journal.pone.0175071 (PMC5383228; doi:10.1371/journal.pone.0175071)
Supplement: S1 Appendix — (PDF) [file pone.0175071.s001.pdf]

S1 Appendix. Details on environmental and genetic variables from the HUNT data

Genotyping

DNA was extracted from peripheral blood leucocytes from EDTA whole blood or blood clots using MasterPure DNA Purification kit (Medinor) or Gentra Purgene blood kit (QIAGEN Science, MD, USA). The procedure was done manually, mainly as described by the manufacturer. DNA concentrations were determined at HUNT biobank by use of PicoGreen dsDNA Quantitation (Life Technologies, Grand Island, NY, USA) where every 8th sample in addition was measured by Nanodrop quantification (Nanodrop Products, Wilmington, DE, USA). Genotyping was performed at HUNT biobank using 7900HT Fast Real-Time PCR System with predesigned TaqMan SNP Genotyping Assays (Life Technologies/ Thermo Fisher Scientific) specified for the two SNPs. Two positive and two negative controls were included on each 384-well sample trays.

The frequencies of the genotypes of FTO (rs9939609) and MC4R (rs17782313) in the extreme phenotype sample are presented in Table A1.

	20-40 years		40-60 years		60-80 years	
	Men	Women	Men	Women	Men	Women
FTO genotype TT	669	903	1021	1297	610	683
FTO genotype TA	1000	1373	1531	1811	967	945
FTO genotype AA	360	464	529	686	348	409
MC4R genotype TT	1096	1486	1681	2986	1010	1133
MC4R genotype TC	815	1051	1184	1458	778	737
MC4R genotype CC	117	203	216	250	137	167

Table A1: Number of individuals with each genotype of the FTO and MC4R SNPs in each age and gender group, for individuals with WHR in the upper and lower quartiles or BMI $\geq$ 35kg/m^2.

Non-genetic variables

Height and weight were measured by trained nurses with the participants wearing light clothes and no shoes; height was measured to the nearest centimetre, and weight to the nearest 0.5 kg. Waist and hip circumferences were measured to the nearest centimetre. Waist was measured at the height of the umbilicus, and hip circumference over the widest part of the hip. Non-genetic explanatory variables were constructed from HUNT3 questionnaire answers on physical exercise, diet and smoking.

The physical activity index was summarized based on questions 32, 33, 34 and 36 in Questionnaire 1 of the HUNT3 study [1]. The questions, with corresponding answer categories, were as follows:

- Q32: How often do you exercise? (on average)
  Options: (a) Never, (b) Less than once a week, (c) Once a week, (d) 2-3 times a week, (e) Nearly every day
- Q33: How hard to you exercise?
  Options: (a) I take it easy, not getting out of breath or break a sweat, (b) I push myself until I am out of breath and break a sweat, (c) I practically exhaust myself

- Q34: How long do you exercise?
  Options: (a) Less than 15 minutes, (b) 15-29 minutes, (c) 30 min – 1 hour, (d) More than one hour

- Q36: Approximately how many hours do you sit still during an average day? (include work hours and leisure time)

For each question we gave a score of 0, 1 or 2 according to increasing levels of physical activity. The scores were defined as follows; Q23: 0 for (a) or (b), 1 for (c), 2 for (d) or (e); Q33: 0 for (a), 1 for (b), 2 for (c); Q34: 0 for (a) and (b), 1 for (c), 2 for (d); Q36: 0 for more than six hours, 1 for between four and six hours, 2 for less than 4 hours. The physical activity (PA) index was defined as a sum of the scores for each question, weighing each question equally. The physical activity index was treated as a continuous covariate in our regression models, i.e. we assumed that on average, WHR or BMI increased linearly according to the PA index. This assumption is not obvious, and we initially treated the PA index as a categorical variable and then observed that the estimated effects were approximately linear.

The variable for artificially sweetened beverages was retrieved from question 25 in Questionnaire 1 of HUNT3 [1]:

- Q25: How many glasses do you usually drink of soda/lemonade without sugar?
  Options: (a) 0 glasses, (b) 1-6 glasses per week, (c) 1 glass per day, (d) 2-3 glass per day, (e) 4 glasses or more a day.

The answer categories were recoded into glasses per week (0, 3.5, 7, 17.5, 28). We note that natural sugar-free fruit juice was asked for in a separate question so we would expect participants to interpret Q25 as referring to artificially sweetened beverages.

We summarized smoking into the variable pack years, which is the number of packs of cigarettes smoked daily (20 cigarettes in one pack), multiplied by the number of years a person has been smoking. We constructed this variable using question 21a in Questionnaire 1 of the HUNT3 study [1]:

- Q21a part 1: How many cigarettes do you (or did you use to) smoke daily?

- Q21a part 2: How old were you when you started smoking?

- Q21a part 3: If you used to smoke daily, how old were you when you stopped smoking?

The number of pack years is then difference in age between Q21a part 2 and Q21a part 3, multiplied by the daily number of packs (answer to Q21a part 1 divided by 20).

References

[1] Questionnaires from the HUNT studies. `http://www.ntnu.no/hunt/sp-rreskjemaer`.
