# Supplementary material for: Assessing gene-environment interaction effects of FTO, MC4R and lifestyle factors on obesity using an extreme phenotype sampling design: Results from the HUNT study
Source: PLoS One. 2017 Apr 6;12(4):e0175071. doi: 10.1371/journal.pone.0175071 (PMC5383228; doi:10.1371/journal.pone.0175071)
Supplement: S2 Appendix — (PDF) [file pone.0175071.s002.pdf]

S2 Appendix. Statistical methods for the extreme phenotype sampling design

By the extreme phenotype sampling design chosen for this study, the genetic variables were observed for about half of the HUNT3 population, while non-genetic explanatory variables and traits were observed for the entire population. We chose a missing covariate likelihood approach for modeling extreme sampling data. A similar approach was taken by Huang and Lin [2] for models with one genetic covariate and no non-genetic covariates. A likelihood that ignores the missing-mechanism, i.e. the specific model for the missing structure in the data, can be used for our extreme phenotype sampling model because the missing data are missing at random (MAR). The genetic covariates are MAR because the missingness does not depend on the unobserved variables themselves, but rather on observed trait (WHR and BMI). Our derivation of the following likelihood is based on Little and Rubin [3, Chapter 6.2], and further developments and general results for EPS data analysis will be presented in future research by the first author of this paper.

We consider linear regression models of the form

$$Y_i = \alpha + \mathbf{x}_{\mathrm{e}i}^{\mathrm{T}}\boldsymbol{\beta}_{\mathrm{e}} + \mathbf{x}_{\mathrm{g}i}^{\mathrm{T}}\boldsymbol{\beta}_{\mathrm{g}} + \varepsilon_i,$$

where $\varepsilon_i \sim \mathcal{N}(0, \sigma^2)$, $i = 1, \ldots, N$, Y_i denotes a phenotype or trait (WHR, BMI), $\mathbf{x}_{\mathrm{e}i}$ are the environmental covariates (age groups, physical activity index, artificially sweetened beverages and smoking) and $\mathbf{x}_{\mathrm{g}i}$ are the genetic covariates (FTO and MC4R). By the extreme phenotype sampling design applied in our study, $\mathbf{x}_{\mathrm{g}i}$ is missing for approximately half of the sample. We define the set of indexes $\mathcal{C}$ such that for all samples $i \in \mathcal{C}$, the trio $(y_i, \mathbf{x}_{\mathrm{e}i}, \mathbf{x}_{\mathrm{g}i})$ is observed, while for the samples $i \notin \mathcal{C}$, the pair $(y_i, \mathbf{x}_{\mathrm{e}i})$ is observed and $\mathbf{x}_{\mathrm{g}i}$ is missing. By the linear regression model we have

$$f_{Y|\mathbf{X}_{\mathrm{g}}=\mathbf{x}_{\mathrm{g}i}}(y_i; \mathbf{x}_{\mathrm{e}i}, \alpha, \boldsymbol{\beta}_{\mathrm{e}}, \boldsymbol{\beta}_{\mathrm{g}}, \sigma) = \phi\left(\frac{y_i - (\alpha + \mathbf{x}_{\mathrm{e}i}^{\mathrm{T}}\boldsymbol{\beta}_{\mathrm{e}} + \mathbf{x}_{\mathrm{g}i}^{\mathrm{T}}\boldsymbol{\beta}_{\mathrm{g}})}{\sigma}\right)$$

where $\phi()$ is the density of the standard normal distribution. More elaborate regression models were used for interaction analysis, see main article. The likelihood that ignores the missing-mechanism is given by

$$\prod_{i\in\mathcal{C}} f_{Y|\mathbf{X}_{\mathrm{g}}=\mathbf{x}_{\mathrm{g}i}}(y_i; \mathbf{x}_{\mathrm{e}i}, \alpha, \boldsymbol{\beta}_{\mathrm{e}}, \boldsymbol{\beta}_{\mathrm{g}}, \sigma) f_{\mathbf{X}_{\mathrm{g}}}(\mathbf{x}_{\mathrm{g}i}) \prod_{i\notin\mathcal{C}} \sum_{\mathbf{x}_{\mathrm{g}}\in\mathcal{X}_{\mathrm{g}}} f_{Y|\mathbf{X}_{\mathrm{g}}=\mathbf{x}_{\mathrm{g}}}(y_i; \mathbf{x}_{\mathrm{e}i}, \alpha, \boldsymbol{\beta}_{\mathrm{e}}, \boldsymbol{\beta}_{\mathrm{g}}, \sigma) f_{\mathbf{X}_{\mathrm{g}}}(\mathbf{x}_{\mathrm{g}}),$$

where we have assumed that the distribution of $\mathbf{X}_{\mathrm{g}}$ (FTO and MC4R) is independent of the environmental covariates $\mathbf{x}_{\mathrm{e}}$; age, physical activity, diet, smoking. If this assumption is wrong, then false positive over-all genetic effects (or gene-environment interaction effects) can occur because the effect of the environmental variable is mediated through the genetic variable. We verified the assumption of independence by testing for independence between the SNPs and the covariates using all individuals with observed genetic variants (Table A1). Furthermore, we considered models for over-all genetic effects with assumed dependencies between genetic variants and environmental covariates and found minimal changes in p-values and estimated effect sizes; an example is shown in Table A2. We can then be quite certain that the findings in our study are not false positive results due to confounding between genetic variants and environmental covariates.

In our analysis the genetic variable was two-dimensional $\mathbf{X}_{\mathrm{g}} = (X_{\mathrm{FTO}}, X_{\mathrm{MC4R}})$. We assumed that the distributions of the genotypes of FTO and MC4R were independent (the two SNPs are located on different chromosomes) and multinomially distributed, taking

Gender	SNP	Age group	ASB	PA	PCYRS
Men	FTO	0.29	0.21	0.70	0.27
Men	MC4R	0.75	0.96	0.53	0.33
Women	FTO	0.08	0.02	0.08	0.30
Women	MC4R	0.46	0.93	0.78	0.02

Table A1: P-values from tests of independence between SNPs and age groups in 5 year intervals (chi-squared test), and between SNPs and continuous covariates (ANOVA with continuous covariate as response and SNP as covariate); artificially sweetened beverages (ASB), physical activity levels (PA), and pack years of smoking (PCYRS). None of the p-values of these 8 tests have p-values below the Bonferroni-adjusted significance threshold $0.05/16 = 0.003125$.

Trait	Age Group	Gender	FTO a1	FTO a2	MC4R a1	MC4R a2
WHR	20-40	Women	0.00417	0.00458	0.00141	0.00128
WHR	20-40	Men	0.00801	0.00676	0.16052	0.13812

Table A2: P-values from tests of over-all effect of SNPs on WHR, first analysis (a1) assuming independence between the genotype distribution and physical activity and second analysis (a2) assuming dependence.

values 0, 1 and 2, with (unknown) probabilities p_0, p_1, p_2 where $p_2 = 1 - p_0 - p_1$ (different parameters for FTO and MC4R). Then the sample space of $\mathbf{X}_\mathrm{g}$ is

$$\mathcal{X}_\mathrm{g} = \{(0,0),(0,1),(0,2),(1,0),(1,1),(1,2),(2,0),(2,1),(2,2)\},$$

and the probability mass function of $\mathbf{X}_\mathrm{g}$ is

$$f_{\mathbf{X}_\mathrm{g}}(\mathbf{x}_{\mathrm{g}i}) = f_{X_\mathrm{FTO}}(x_{\mathrm{FTO}i}) f_{X_\mathrm{MC4R}}(x_{\mathrm{MC4R}i}).$$

Maximum likelihood estimates were obtained by numerical optimization of the log-likelihood. The Hessian matrix H was also obtained from the optimization, and we estimated the information matrix by $\hat{I} = -H$. We obtained asymptotic confidence intervals for any coefficient β of the linear regression model by

$$\left[\hat{\beta} - z_{\alpha/2}\sqrt{(\hat{I}^{-1})_{\beta,\beta}}, \hat{\beta} + z_{\alpha/2}\sqrt{(\hat{I}^{-1})_{\beta,\beta}}\right],$$

where $z_{\alpha/2}$ is the critical value of the standard normal distribution. We compared these asymptotic confidence intervals to bootstrap intervals and found little discrepancy. The score test was implemented for main- and longitudinal effects when the null model included only completely observed non-genetic covariates. The likelihood ratio test was developed and implemented for interaction effects when genetic covariates with missing values were present in the null model. Both the score and likelihood ratio test statistics are asymptotically χ^2_1 distributed when we test one parameter at a time, and p-values were found accordingly. Derivations and details on these tests will be presented in future research by the first author of this paper. For computational code for maximum likelihood estimation and hypothesis testing, please contact the first author or download our R-package [1].

References

[1] Thea Bjørnland. Github repository for R-package Extremesampling. https://github.com/theabjorn/extremesampling, 2016.

[2] BE Huang and DY Lin. Efficient association mapping of quantitative trait loci with selective genotyping. *The American Journal of Human Genetics*, 80(3):567–576, 2007.

[3] Roderick J Little and Donald B Rubin. *Statistical analysis with missing data.* John Wiley & Sons, 2nd edition, 2002.
