# Supplementary material for: Assessing gene-environment interaction effects of FTO, MC4R and lifestyle factors on obesity using an extreme phenotype sampling design: Results from the HUNT study
Source: PLoS One. 2017 Apr 6;12(4):e0175071. doi: 10.1371/journal.pone.0175071 (PMC5383228; doi:10.1371/journal.pone.0175071)
Supplement: S3 Appendix — (PDF) [file pone.0175071.s003.pdf]

S3 Appendix. Power simulation study

We performed a simulation study based on data from the female 20-40 year age group to evaluate the power of the extreme sampling design, compared to a random sampling design, for selecting individuals for genotyping. The sample size for this group was 4817. The simulation was set up as follows. Covariates age (5-year intervals), physical activity index, diet (artificially sweetened beverages), smoking (pack years) and FTO (for extreme phenotype individuals) were taken from the real data set. The maximum likelihood estimates of parameters of the linear model

$$\begin{aligned}\mathrm{WHR} = \alpha &+ \beta_{\mathrm{age}} x_{\mathrm{age}} + \beta_{\mathrm{PCYR}} x_{\mathrm{PCYR}} + \beta_{\mathrm{ASB}} x_{\mathrm{ASB}} + \beta_{\mathrm{PA}} x_{\mathrm{PA}} \\ &+ \beta_{\mathrm{FTO}} x_{\mathrm{FTO}} + \beta_{\mathrm{PA*FTO}} x_{\mathrm{PA}} x_{\mathrm{FTO}} + \varepsilon,\end{aligned}$$

were obtained by numerical optimization of the extreme sampling likelihood. The parameter estimates were:

α		0.8049899748
β_{age}	Age group 2	0.0175029650
	Age group 3	0.0286825271
	Age group 4	0.0231866438
β_{PCYR}		0.0019860431
β_{ASB}		0.0013601182
β_{PA}		-0.0003809899
β_{FTO}		0.0174521801
$\beta_{\mathrm{PA*FTO}}$		-0.0028787107
σ		0.07144828

A new genetic covariate (x_g) was simulated by drawing the values 0, 1 or 2, with frequencies $p_0 = 0.36$, $p_1 = 0.48$ and $p_2 = 0.16$, for each of the $N = 4817$ individuals. These would be the frequencies of a SNP with minor allele frequency 0.4 (approximately the minor allele frequency of the FTO SNP). A new response y_i was generated by sampling from a normal distribution with mean

$$\mu_i = \alpha + \beta_{\mathrm{age}} x_{\mathrm{age}i} + \beta_{\mathrm{PCYR}} x_{\mathrm{PCYR}i} + \beta_{\mathrm{ASB}} x_{\mathrm{ASB}i} + \beta_{\mathrm{PA}} x_{\mathrm{PA}i} + \beta_{\mathrm{FTO}} x_{gi} + \beta_{\mathrm{PA*FTO}} x_{\mathrm{PA}i} x_{gi},$$

and variance σ^2, for $i = 1, \ldots, N$. In such a simulated data set there are no missing observations of x_g, and we tested the null hypothesis $H_0 : \beta_{\mathrm{PA*FTO}} = 0$ using standard methods. Repeating this $R = 10000$ times, the power estimate was the fraction of tests that yielded a p-value below 0.05. For the full sample, the estimated power was 84% at significance level 0.05. In these $R = 10000$ simulated data-sets, we also selected an extreme sample (genetic variant x_g only known for individuals in the lower and upper quartiles of simulated response y) and a random sample (genetic variant x_g only known for a random sample of half the sample size), and in both of these tested the null hypothesis using appropriate methods. Power estimates were then 80% for the extreme samples, and 56% for the random samples.
