# Supplementary material for: Assessing gene-environment interaction effects of FTO, MC4R and lifestyle factors on obesity using an extreme phenotype sampling design: Results from the HUNT study
Source: PLoS One. 2017 Apr 6;12(4):e0175071. doi: 10.1371/journal.pone.0175071 (PMC5383228; doi:10.1371/journal.pone.0175071)
Supplement: S1 Table — Estimated over-all effects (βSNP) with 95% confidence intervals for men and women in age groups 20–40 years, 40–60 years and 60–80 years. FDR-adjusted p-values for testing H0: βSNP = 0 against H1: βSNP ≠ 0. (PDF) [file pone.0175071.s004.pdf]

S1 Table. Over-all genetic effects.

Trait	SNP	Age group	Gender	β_{SNP}	95% CI	Adjusted p-value
WHR	FTO	20-40 years	Men	0.0045	0.0013, 0.0077	0.048
			Women	0.0048	0.0016, 0.008	0.029
		40-60 years	Men	0.0036	0.0015, 0.0057	0.014
			Women	0.0033	0.0011, 0.0055	0.024
		60-80 years	Men	0.0022	-0.00066, 0.0051	0.36
			Women	0.0019	-0.0014, 0.0051	0.50
	MC4R	20-40 years	Men	0.0028	-0.00095, 0.0065	0.40
			Women	0.0058	0.0023, 0.0092	0.017
		40-60 years	Men	0.0024	4e-05, 0.0047	0.15
			Women	0.0033	0.00077, 0.0057	0.048
		60-80 years	Men	0.0004	-0.0028, 0.0036	1
			Women	0.0062	0.0025, 0.0099	0.015
BMI	FTO	20-40 years	Men	0.37	0.15, 0.59	0.015
			Women	0.53	0.3, 0.76	0.00023
		40-60 years	Men	0.32	0.17, 0.47	0.00071
			Women	0.49	0.33, 0.66	6.7e-07
		60-80 years	Men	0.24	0.05, 0.43	0.072
			Women	0.25	0.02, 0.48	0.12
	MC4R	20-40 years	Men	0.24	-0.015, 0.49	0.20
			Women	0.63	0.38, 0.88	5.2e-05
		40-60 years	Men	0.33	0.17, 0.5	0.0019
			Women	0.24	0.044, 0.43	0.048
		60-80 years	Men	-0.0028	-0.21, 0.21	1
			Women	0.45	0.19, 0.71	0.014

Estimated over-all effects (β_{SNP}) with 95% confidence intervals for men and women in age groups 20-40 years, 40-60 years and 60-80 years. FDR-adjusted p-values for testing H_0: $\beta_{SNP} = 0$ against H_1: $\beta_{SNP} \neq 0$
