# Supplementary material for: Assessing gene-environment interaction effects of FTO, MC4R and lifestyle factors on obesity using an extreme phenotype sampling design: Results from the HUNT study
Source: PLoS One. 2017 Apr 6;12(4):e0175071. doi: 10.1371/journal.pone.0175071 (PMC5383228; doi:10.1371/journal.pone.0175071)
Supplement: S2 Table — Estimated longitudinal effects (βSNP) with 95% confidence intervals for men and women in age groups 30–40 years (individuals younger than 30 years in HUNT3 did not participate in HUNT2), 40–60 years and 60–80 years. FDR-adjusted p-values for testing H0: βSNP = 0 against H1: βSNP ≠ 0. (PDF) [file pone.0175071.s005.pdf]

S2 Table. Longitudinal genetic effects.

Trait	SNP	Age group	Gender	β_{SNP}	95% CI	Adjusted p-value
WHR	FTO	30-40 years	Men	0.0023	-0.0029, 0.0075	0.71
			Women	0.0046	-0.0014, 0.011	0.36
		40-60 years	Men	0.0015	-0.00095, 0.0039	0.5
			Women	0.0026	3.8e-05, 0.0051	0.15
		60-80 years	Men	-0.00073	-0.0037, 0.0023	0.86
			Women	-0.00033	-0.0036, 0.0029	1
	MC4R	30-40 years	Men	0.00076	-0.0055, 0.0071	1
			Women	0.0074	0.00092, 0.014	0.11
		40-60 years	Men	0.00017	-0.0025, 0.0028	1
			Women	0.00063	-0.0023, 0.0035	0.86
		60-80 years	Men	0.00032	-0.003, 0.0036	1
			Women	0.0033	-0.00032, 0.0069	0.21
BMI	FTO	30-40 years	Men	-0.091	-0.33, 0.15	0.74
			Women	0.091	-0.18, 0.36	0.78
		40-60 years	Men	0.14	0.045, 0.23	0.029
			Women	0.11	-0.0065, 0.22	0.2
		60-80 years	Men	0.0095	-0.1, 0.12	1
			Women	-0.0023	-0.13, 0.13	1
	MC4R	30-40 years	Men	0.096	-0.2, 0.39	0.78
			Women	0.32	0.032, 0.62	0.11
		40-60 years	Men	0.041	-0.062, 0.14	0.73
			Women	-0.047	-0.18, 0.083	0.78
		60-80 years	Men	-0.17	-0.3, -0.043	0.048
			Women	0.086	-0.06, 0.23	0.5

Estimated longitudinal effects (β_{SNP}) with 95% confidence intervals for men and women in age groups 30-40 years (individuals younger than 30 years in HUNT3 did not participate in HUNT2), 40-60 years and 60-80 years. FDR-adjusted p-values for testing H_0: $\beta_{SNP} = 0$ against H_1: $\beta_{SNP} \neq 0$.
