# Supplementary material for: Assessing gene-environment interaction effects of FTO, MC4R and lifestyle factors on obesity using an extreme phenotype sampling design: Results from the HUNT study
Source: PLoS One. 2017 Apr 6;12(4):e0175071. doi: 10.1371/journal.pone.0175071 (PMC5383228; doi:10.1371/journal.pone.0175071)
Supplement: S3 Table — Average differences in WHR and BMI between HUNT3 and HUNT2 for men and women in three age groups. Positive differences indicate increase from HUNT2 to HUNT3. (PDF) [file pone.0175071.s006.pdf]

S3 Table. Trait differences HUNT2 and HUNT3

Age (HUNT3)	Gender	Sample size	Average difference in WHR	Average difference in BMI
30-40	Men	993	0.06	2.24
	Women	1505	0.08	1.89
40-60	Men	5216	0.05	1.42
	Women	6397	0.08	1.65
60-80	Men	3639	0.05	0.75
	Women	3660	0.09	0.91

Average differences in WHR and BMI between HUNT3 and HUNT2 for men and women in three age groups. Positive differences indicate increase from HUNT2 to HUNT3.
